# Neuroimaging abnormalities associated with immunotherapy responsiveness in Down syndrome regression disorder

**DOI:** 10.1002/acn3.52023

**Published:** 2024-02-20

**Authors:** Jonathan D. Santoro, Mellad M. Khoshnood, Saba Jafarpour, Lina Nguyen, Natalie K. Boyd, Benjamin N. Vogel, Ryan Kammeyer, Lina Patel, Melanie A. Manning, Angela L. Rachubinski, Robyn A. Filipink, Nicole T. Baumer, Stephanie L. Santoro, Catherine Franklin, Benita Tamrazi, Kristen W. Yeom, Gordon Worley, Joaquin M. Espinosa, Michael S. Rafii

**Affiliations:** ^1^ Division of Neuroimmunology, Department of Pediatrics Children's Hospital Los Angeles Los Angeles California USA; ^2^ Department of Neurology Keck School of Medicine of the University of Southern California Los Angeles California USA; ^3^ Department of Neurology Children's Hospital Colorado Aurora Colorado USA; ^4^ Department of Pharmacology, Linda Crnic Institute for Down Syndrome University of Colorado Anschutz Medical Campus Aurora Colorado USA; ^5^ Department of Genetics Stanford University School of Medicine Palo Alto California USA; ^6^ Division of Child Neurology, Department of Pediatrics University of Pittsburgh School of Medicine Pittsburgh Pennsylvania USA; ^7^ Division of Developmental Medicine, Department of Pediatrics Boston Children's Hospital, Harvard Medical School Boston Massachusetts USA; ^8^ Department of Neurology Boston Children's Hospital, Harvard Medical School Boston Massachusetts USA; ^9^ Genetics and Metabolism Division Massachusetts General Hospital for Children Boston Massachusetts USA; ^10^ Department of Pediatrics Harvard Medical School Boston Massachusetts USA; ^11^ Mater Research Institute‐UQ The University of Queensland Brisbane Queensland Australia; ^12^ Department of Radiology Children's Hospital Los Angeles and Keck School of Medicine of the University of Southern California Los Angeles California USA; ^13^ Department of Radiology Stanford University School of Medicine Palo Alto California USA; ^14^ Department of Pediatrics Duke University School of Medicine Durham North Carolina USA; ^15^ Alzheimer's Therapeutic Research Institute Keck School of Medicine of the University of Southern California San Diego California USA

## Abstract

**Objective:**

To determine the prevalence of neuroimaging abnormalities in individuals with Down syndrome regression disorder (DSRD) and evaluate if neuroimaging abnormalities were predictive of therapeutic responses.

**Methods:**

A multicenter, retrospective, case–control study which reviewed neuroimaging studies of individuals with DSRD and compared them to a control cohort of individuals with Down syndrome (DS) alone was performed. Individuals aged 10–30 years and meeting international consensus criteria for DSRD were included. The presence of T1, T2/FLAIR, and SWI signal abnormalities was reviewed. Response rates to various therapies, including immunotherapy, were evaluated in the presence of neuroimaging abnormalities.

**Results:**

In total, 74 individuals (35%) had either T2/FLAIR and/or SWI signal abnormality compared to 14 individuals (12%) without DSRD (*p* < 0.001, 95%CI: 2.18–7.63). T2/FLAIR signal abnormalities were not appreciated more frequently in individuals with DSRD (14%, 30/210) than in the control cohort (9%, 11/119) (*p* = 0.18, OR: 1.63, 95%CI: 0.79–3.40). SWI signal abnormalities were appreciated at a higher frequency in individuals with DSRD (24%, 51/210) compared to the control cohort (4%, 5/119) (*p* < 0.001, OR: 7.31, 95%CI: 2.83–18.90). T2/FLAIR signal abnormalities were localized to the frontal (40%, 12/30) and parietal lobes (37%, 11/30). SWI signal abnormalities were predominantly in the bilateral basal ganglia (94%, 49/52). Individuals with DSRD and the presence of T2/FLAIR and/or SWI signal abnormalities were much more likely to respond to immunotherapy (*p* < 0.001, OR: 8.42. 95%CI: 3.78–18.76) and less likely to respond to benzodiazepines (*p* = 0.01, OR: 0.45, 95%CI: 0.25–0.83), antipsychotics (*p* < 0.001, OR: 0.28, 95%CI: 0.11–0.55), or electroconvulsive therapy (*p* < 0.001, OR: 0.12; 95%CI: 0.02–0.78) compared to individuals without these neuroimaging abnormalities.

**Interpretation:**

This study indicates that in individuals diagnosed with DSRD, T2/FLAIR, and SWI signal abnormalities are more common than previously thought and predict response to immunotherapy.

## Introduction

Down syndrome (DS), a genetic condition most often caused by trisomy of chromosome 21, and is one of the most common genetic disorders, occurring in approximately 1 in every 700 live births in the United States.^1^, ^2^ Individuals with DS have well‐phenotyped neurologic and psychiatric disease, although acute and subacute neurocognitive regression of unclear etiology in individuals considered too young to develop Alzheimer's disease (AD) and too old to develop autism spectrum disorder (ASD) has been increasingly reported. Down syndrome regression disorder (DSRD) and has principally been reported in individuals with DS aged 10–30 years.^3^, ^4^ Symptoms include an acute or subacute deterioration in adaptive skills, behavioral regulation, cognition, communication, language, and a loss of previously acquired developmental milestones and skills.^3^, ^4^, ^5^, ^6^, ^7^ Other symptoms can include bradykinesia, catatonia, psychiatric symptoms, and rapid onset insomnia.^5^, ^7^, ^8^ The symptoms of DSRD can be profound and significantly impact the quality of life and autonomy of both individuals with DS as well as impacting their families and caregivers.

The search for neurodiagnostic biomarkers of disease in DSRD remains critical given the wide array of therapeutics available for this condition. An earlier study has indicated that individuals with neuroimaging abnormalities are more likely to respond to immunotherapy.^7^ Previously, reports of T2 signal prolongations in the frontal and temporal lobe as well as SWI signal abnormality in the basal ganglia have been reported.^7^, ^9^ However, detailed neuroradiologic investigation has not been reported.

This study sought to retrospectively assess the prevalence of neuroimaging abnormalities in individuals with DSRD and determine if the presence of neuroimaging abnormalities were associated with a higher response rate to particular therapeutic interventions.

## Methods

### Study approval and data availability

IRB approval was obtained for this study. IRB approval for a waiver of consent was authorized for de‐identified review of individuals at the host institution (Children's Hospital Los Angeles [CHLA]). Individuals evaluated outside the host institution had caregivers and/or guardians' consent to a HIPPA waiver and record release for clinical and research review.

Anonymized data are available to qualified researchers upon request to the corresponding author and IRB approval.

### Participants and study design

Individuals were identified by having participated in either in‐person or telehealth‐based clinical consultation for DSRD at multiple sites between July 1, 2019, and June 1, 2023. To be included in this study, external clinicians and individuals/guardians had to have been referred for clinical evaluation or second opinion at the host site (CHLA) and consented to record release and upload of neuroimaging. Inclusion criteria included age between 10 and 30 years at symptom onset, diagnosis of either possible or probable DSRD per expert consensus guidelines,^10^ and obtainment of neurodiagnostic studies (EEG, MRI, and lumbar puncture [LP]). All neuroimaging studies had to be on a 3 tesla (T) scanner. Images could have been obtained at any time point during the work up of DSRD but had to be obtained prior to use of any immunotherapy. Each individual's diagnosis was confirmed by an arbiter with no knowledge of the case (NKB) retrospectively through chart review. The arbiter was blinded to the results of neuroimaging abnormalities and responses to treatment. Exclusion criteria included age less than 10 years or greater than 30 years at the time of symptom onset, active cardiac and/or pulmonary disease, frequent infection (defined as more than two infections requiring antibiotics or antivirals per year), a history of neoplasia or receipt of chemotherapy or radiation, active or a history of epilepsy (excluding febrile seizure), use of electroconvulsive therapy (ECT), and/or immunotherapy not related to DSRD at the time of diagnosis. Individuals with a history of cardiac surgery or non‐atrial septal defect (ASD), ventricular septal defect (VSD), or patent foramen ovale (PFO) (e.g., tetralogy of Fallot) cardiac pathology were excluded from this study as this could influence neuroimaging abnormalities. Individuals with prior neurosurgical intervention of any type were excluded as well.

Demographic data, including medical/surgical history, and results of clinical and diagnostic investigations were collected from clinical documentation and entered into a de‐identified form in a RedCAP database. At the initial evaluation visit, all caregivers (or patients when able) were asked to identify any potential triggers that were associated with the onset of symptoms. These were included as potentially contributory to each case if they preceded the onset of DSRD symptoms by 12 weeks or less. All individuals in this study received a neuropsychiatric inventory (NPI‐Q), Bush‐Francis Catatonia Rating Scale (BFCRS), timed 25‐foot walk (25FW), and Clinical Global Improvement Scale (CGI), as part of each visit.

Individuals with DSRD were compared to individuals with only DS who had an MRI performed between January 1, 2012 and June 1, 2021. A 2‐year gap between the last date of MRI review in the control and experimental cohorts was utilized to ensure that individuals in the control cohort did not go on to subsequently develop DSRD. Neuroimaging data was obtained from two institutional retrospective databases of persons with DS. Inclusion in the comparator group only required a prior diagnosis of DS and neuroimaging obtained at any time point prior to analysis outside of the neonatal period (greater than 30 days). The same exclusion criteria were applied. Indication for neuroimaging was extracted from ICD‐9 or ICD‐10 codes used for study authorization and/or chart review.

### Imaging requirements and quality control

MRIs were performed on 3T scanner with a 32‐channel head coil. Devices could be of either Phillips, General Electric, or Siemen's brand. For studies done outside the primary site, a research coordinator confirmed these parameters independently with cases not meeting parameters requiring exclusion. The minimum sequences obtained for inclusion were T1, T2, fluid‐attenuated inversion recovery (FLAIR), and susceptibility weighted imaging (SWI), although other sequences could have been obtained for clinical purposes. Axial SWI were required to be acquired using the following parameters: TR = 31 ms, TE/ΔTE = 7.2/Δ6.2 ms, flip angle 17°, and slice thickness = 2.0 mm. Axial T2‐weighted imaging were required to be acquired using the following parameters: TR = 4000 ms, TE = 99 ms, flip angle 90°, and slice thickness = 4 mm.

Images were transferred to the host site by either manual compact disc upload or secure Picture Archiving and Communication System (PACS) push. All images were reviewed by two board‐certified neuroradiologists with no knowledge of the clinical history or diagnosis. Reviewers were asked to comment on structural, T2/FLAIR, SWI, and post‐contrast abnormalities. Images of inferior quality of either T1, T2/FLAIR, and/or SWI sequences resulted in exclusion from this study and was at the discretion of any reviewing neuroradiologist. For any individual with multiple neuroimaging studies, the first study performed after the onset of symptoms occurred was used.

### Determination of an abnormal study

1. T1: Abnormalities on T1 sequencing were defined as structural abnormalities of the gray matter, white matter, brainstem, or cerebellum. Definition of hypoplasia of a structure was a region demonstrating volume loss but not structural defects and was assessed subjectively (quantitative volumetry not performed).
2. T2: Abnormalities on T2/FLAIR sequencing were defined as lesions of gray matter or white matter measuring greater than 2 mm in diameter. Imaging abnormalities had to be present on both T2 and FLAIR images and had to be present on a two different field view (axial, sagittal, or coronal) to be defined as abnormal.
3. SWI: Abnormalities on SWI sequences were defined as any signal abnormality of any size or distribution in the gray or white matter structures. Anatomical variants in the cerebrovasculature detected on SWI (e.g., hypoplastic vessel) were not considered abnormal unless they were deemed to be potentially clinically significant (e.g., narrowing of the internal carotid arteries consistent with moyamoya disease).

### Phenotyping abnormalities

Neuroimaging abnormalities, when present, were subsequently characterized by quantity (total number), size (defined by largest diameter and measured in mm), location (frontal, temporal, parietal, or occipital lobes and/or cerebellum and brainstem), structural involvement (gray matter or white matter), and the presence or absence of clustering of lesions. Particular radiographic patterns (e.g., cerebral amyloid angiopathy) were also requested to be assessed if present by the independent reviewers.

### Determination of response

This study evaluated if neuroimaging findings were associated with DSRD clinical response to therapies (antidepressants, antipsychotics, benzodiazepines, ECT, or immunotherapy) commonly used to treat this condition.^5^, ^7^ Clinical response was evaluated using validated metrics including the NPI‐Q,^11^ BFCRS,^12^, ^13^ 25FW,^14^ and CGI.^15^ Clinical response was classified as a greater than 50% improvement in two of four assessment tools at 12 weeks after treatment initiation (compared to baseline, pretreatment score). Responses were coded as a binary response or no response. Individuals who had previously been on therapy prior to obtainment of the NPI‐Q, BFCRS, 25FW, or CGI, and thus had no baseline scoring, had these therapeutic responses excluded. For instance, if an individual had previously been on fluoxetine (antidepressant) prior to the baseline objective assessment, he or she did not have the antidepressant category response tabulated. All subsequent therapeutic trials were calculated.

### Statistical analysis

Descriptive statistics were utilized to report demographic, clinical, and neuroimaging data. These data were subsequently compared between individuals with and without neuroimaging abnormalities using chi‐square (χ^2^) or Fisher's exact tests for categorical variables, and t‐test for continuous variables. Additional mixed‐effects models with similar covariance structure were used including the fixed effects for those with neuroimaging abnormalities to those without. The models further allowed for interaction effects between demographics and neuroimaging abnormalities and therapeutic responses. Inter‐rater reliability on neuroimaging was determined by calculating Cohen's Kappa coefficient. Statistical significance was determined by an alpha less than 0.05.

A univariate logistic regression was used to model the association between neuroimaging abnormalities and demographic, clinical, and therapeutic response variables. Variables which differed substantially by response were entered into an independent multivariable logistic regression model. All analyses were conducted using Stata/MP version‐17.0 (StataCorp. 2021. College Station, TX: StataCorp LLC.).

## Results

In total, 337 patients were identified for inclusion and 247 of these individuals (73%) had neuroimaging with T1, T2, and SWI sequences available on a 3T scanner. An additional 53 individuals (16%) were excluded for having congenital cardiac surgery, 24 individuals (7%) did not have sufficient clinical information available for review, and 13 individuals (4%) had uninterpretable or poor‐quality neuroimaging studies. As such, 210 individuals were included in the analysis. A control cohort of 119 individuals with DS was also identified. Inter‐rater reliability between the two neuroradiologists across all scans was 96.3% (Cohen's Kappa coefficient: 0.83, SE of Kappa: 0.082, 95%CI: 0.69–0.96).

Demographic and clinical data are presented in Table 1. The control cohort had higher rates of prematurity (*p* < 0.001, 95%CI: 0.09–0.37), epilepsy/seizure (*p* < 0.001, 95%CI: 0.02–0.15), and autism (*p* < 0.001, 95%CI: 0.02–0.18) compared to those with DSRD. Individuals with DSRD had higher rates of preceding autoimmune disease compared to controls (*p =* 0.02, 95%CI: 1.11–2.96). The most common autoimmune conditions encountered in the DSRD cohort were Hashimoto's thyroiditis (36%, 30/84), celiac disease (26%, 22/84), autoimmune skin disease (23%, 19/84), and type I diabetes (18%, 15/84). The most common indications for neuroimaging in the control cohort were epilepsy/seizure (42%, 50/119), severe hypotonia (18%, 22/119), focal weakness or spasticity (16%, 19/119), and profound global developmental delay (12%, 14/119).

**Table 1:** Demographic and clinical data.

	DSRD (*n* = 210)	DS w/o DSRD (*n* = 119)	*p* value	95%CI
Sex				
Male	109 (52%)	62 (52%)	0.97	0.63–1.56
Female	101 (48%)	57 (48%)		
Race				
White	151 (72%)	89 (75%)		
Black/African‐American	21 (10%)	8 (7%)		
Asian	18 (9%)	8 (7%)	0.57	0.52–1.44
AI/AN	6 (3%)	2 (2%)		
NH/OPI	2 (1%)	2 (2%)		
Other	12 (6%)	10 (8%)		
Ethnicity				
Hispanic	111 (53%)	72 (60%)	0.18	0.46–1.16
Not Hispanic	99 (47%)	47 (40%)		
Age at MRI (median, IQR)	17 (14–23)	15 (10–22)	0.22	0.32–2.08
History of prematurity	12 (6%)	30 (25%)	**<0.001**	**0.09–0.37**
History of epilepsy/seizure	5 (2%)	36 (30%)	**<0.001**	**0.02–0.15**
History of autoimmune disease	84 (40%)	32 (24%)	**0.02**	**1.11–2.96**
History of autism diagnosis^a^	4 (2%)	29 (24%)	**<0.001**	**0.02–0.18**
Indication for neuroimaging				
DSRD	210 (100%)	0 (0%)		
Developmental regression	0 (0%)	5 (4%)		
Epilepsy/seizure	0 (0%)	50 (42%)	–	–
Severe hypotonia	0 (0%)	22 (18%)		
Focal weakness or spasticity	0 (0%)	19 (16%)		
Profound global developmental delay	0 (0%)	14 (12%)		
Other	0 (0%)	9 (8%)		
Age at symptom onset (median, IQR)	14 (11–18)	–	–	–
Time (months) to symptom nadir	3.0 (2–5)	–	–	–
Potential trigger present	107 (51%)	–	–	–
Type of potential trigger				
Infection (<8 weeks prior)	50 (47%)			
Change in environment	18 (17%)			
Change in residence	13 (12%)	–	–	–
Abuse	3 (3%)			
Medical change/hospitalization	2 (2%)			
Death in family	2 (2%)			
Possible DSRD Criteria met	210 (100%)	1 (1%)^b^	–	–
Probable DSRD Criteria met	144 (69%)	0 (0%)	–	–
Catatonia	164 (78%)	1 (1%)	**<0.001**	**57.2–3093**

Bolded items indicate *p* <0.05.

AI/AN, American Indian or Alaskan Native; CI, confidence interval; DS, Down syndrome; DSRD; Down Syndrome Regression Disorder; IQR, interquartile range; NH/OPI, Native Hawaiian or Other Pacific Islander; w/o, without.

^a^ Excludes prior diagnosis of autism spectrum disorder misdiagnosed for DSRD.

^b^ Patient had definitive autism spectrum disorder from a young age, onset of symptoms outside those of DSRD.

**Figure 1:**
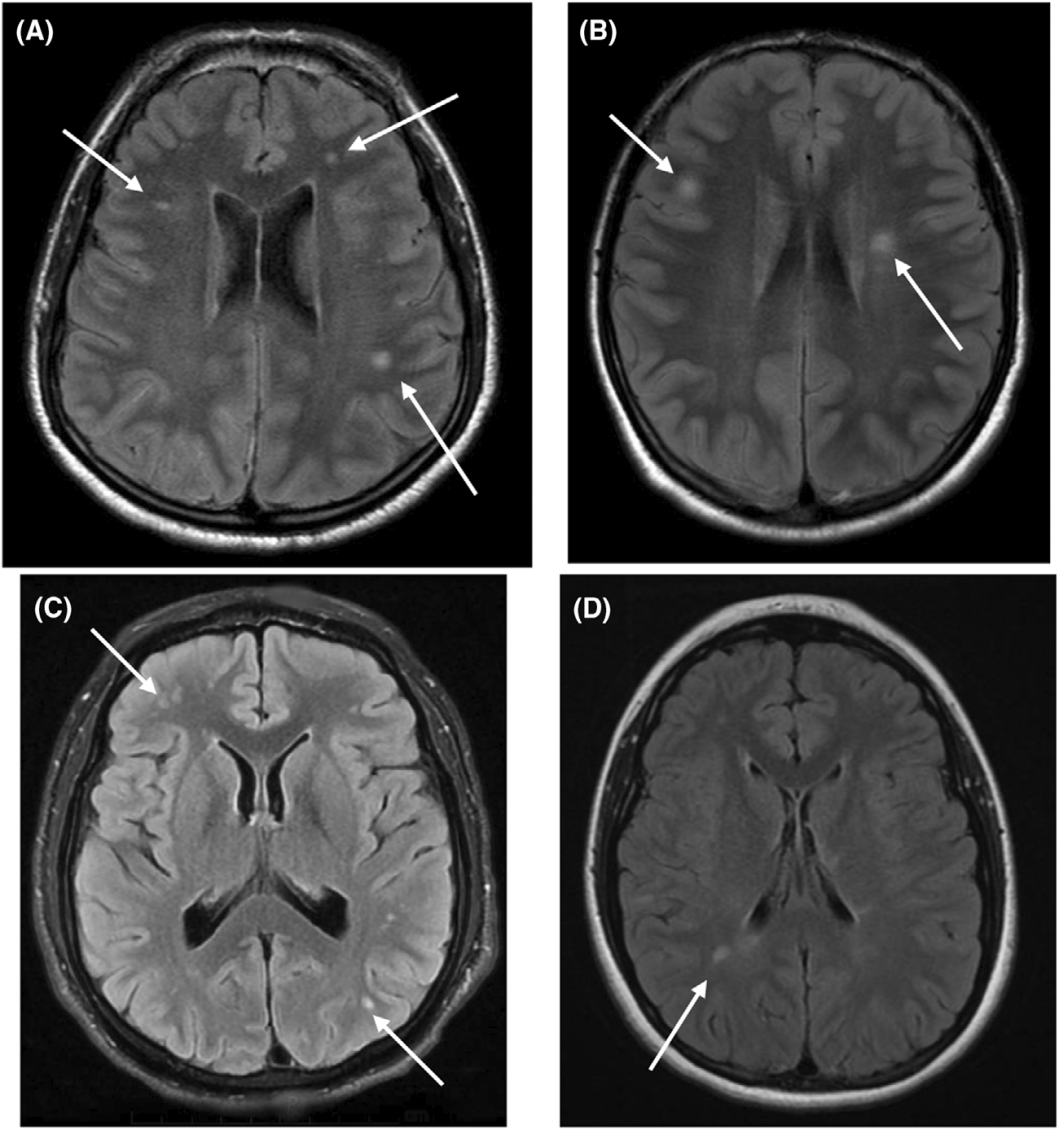
Examples of T2/FLAIR signal abnormalities in individuals DSRD. (A–D) Axial T2/FLAIR sequences demonstrating focal T2 signal prolongation throughout the white matter. White arrows highlight characteristic lesions identified in the white matter.

**Figure 2:**
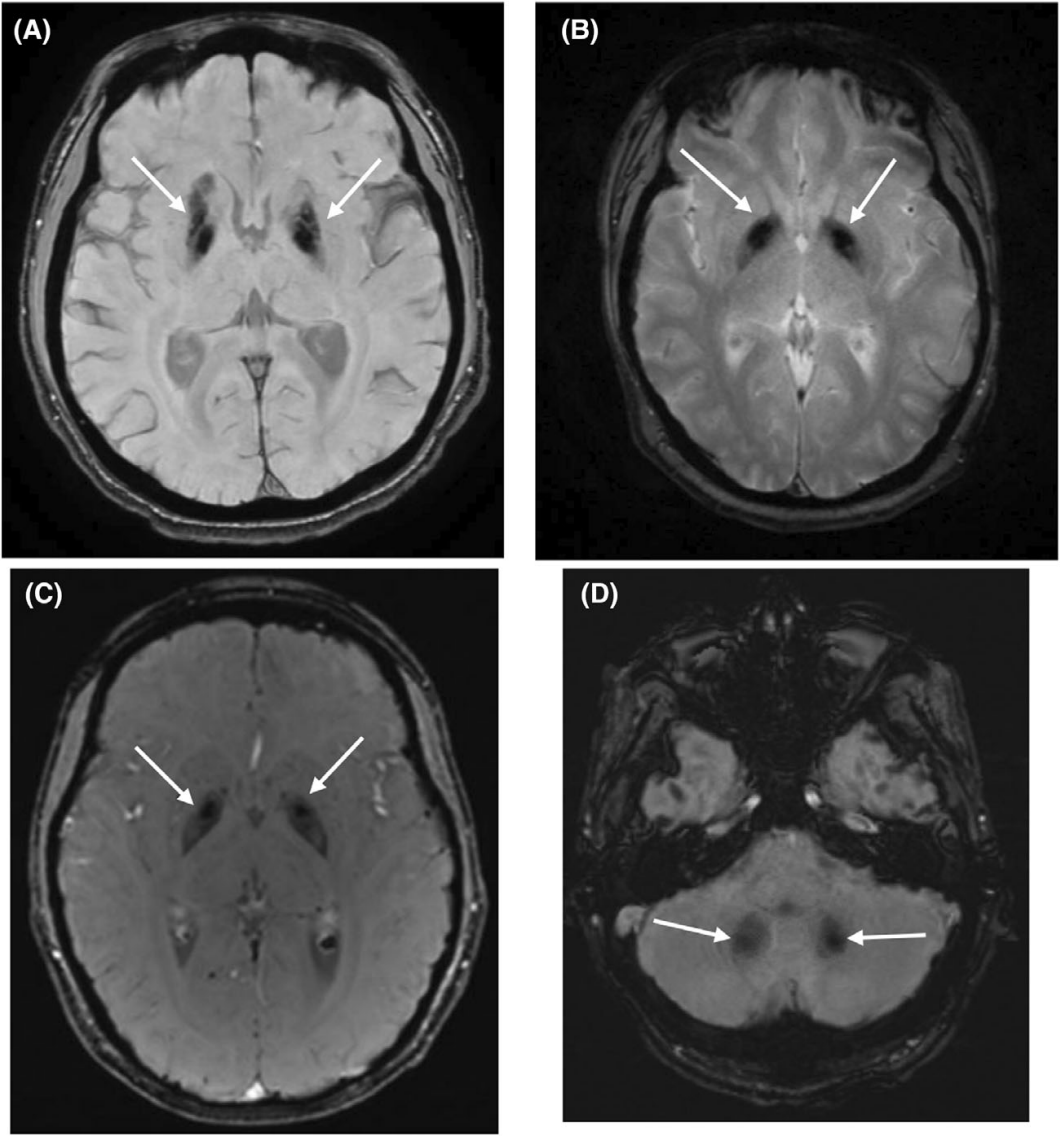
SWI abnormalities in individuals with DSRD (A–D). (A–C) Axial SWI signal abnormality of the bilateral basal ganglia. (D) Axial SWI signal abnormality in the bilateral dentate nuclei. White arrows indicate areas of signal abnormality.

Differences in T1, T2, and SWI sequences in DSRD and control populations are presented in Table 2. There was a low rate of structural T1 abnormalities appreciated in the DSRD cohort (3%, 6/210) compared to the control cohort (27%, 33/119) (*p <* 0.001, OR: 0.08, 95%CI: 0.03–0.19). T2/FLAIR signal abnormalities were not appreciated more frequently in individuals with DSRD (14%, 30/210) than in the control cohort (9%, 11/119) (*p* = 0.18, OR: 1.63, 95%CI: 0.79–3.40). Age (*p* = 0.55, 95%CI: 0.63–2.69) and sex (*p* = 0.74, 95%CI: 0.12–4.69) were not predictive of the presence of T2/FLAIR signal abnormalities in the DSRD cohort. SWI signal abnormalities were appreciated at a higher frequency in individuals with DSRD (24%, 51/210) compared to the control cohort (4%, 5/119) (*p* < 0.001, OR: 7.31, 95%CI: 2.83–18.90). Age (*p* = 0.09, 95%CI: 0.88–1.31) and sex (*p* = 0.87, 95%CI: 0.32–3.47) were not predictive of the presence of SWI signal abnormalities in the DSRD cohort. Representative images of both T2/FLAIR and SWI signal abnormalities are displayed in Figures 1 and 2, respectively.

**Table 2:** Prevalence of imaging abnormalities in individuals with and without DSRD.

	DSRD (*n* = 210)	DS w/o DSRD (*n* = 119)	*p* value	Odds ratio	95%CI
Presence of T1 or structural abnormalities	6 (3%)	33 (27%)	**<0.001**	**0.08**	**0.03–0.19**
Location of T1 or structural abnormalities					
Atrophic cerebellum	3 (50%)	10 (30%)	0.35	2.3	0.39–13.42
Absent corpus collosum	0 (0%)	3 (9%)	0.79	0.67	0.03–14.61
Hypoplastic corpus collosum	1 (17%)	5 (15%)	0.92	1.12	0.12–11.73
Underdeveloped hippocampi	1 (17%)	8 (24%)	0.69	0.63	0.06–6.17
Focal hypoplasia	1 (17%)	7 (21%)	0.80	0.74	0.07–7.43
Presence of T2/FLAIR signal abnormality	30 (14%)	11 (9%)	0.18	1.63	0.79–3.40
Location of T2/FLAIR signal abnormalities					
Frontal lobe	12 (40%)	1 (9%)	0.08	6.68	0.75–59.07
Parietal lobe	11 (37%)	2 (18%)	0.27	2.61	0.47–14.30
Temporal lobe	5 (17%)	3 (27%)	0.45	0.53	0.11–2.74
Occipital lobe	2 (7%)	3 (27%)	0.09	0.19	0.03–1.34
≥2 Lobes involved	8 (27%)	1 (9%)	0.25	3.64	0.40–33.12
White matter	28 (93%)	10 (91%)	0.79	1.40	0.11–17.17
Gray matter	2 (7%)	1 (9%)	0.79	0.71	0.06–8.76
Brainstem	0 (0%)	0 (0%)	–	–	–
Cerebellum	1 (3%)	2 (18%)	0.15	0.16	0.01–1.92
Clustered lesions	8 (4%)	1 (1%)	0.15	4.67	0.58–37.83
Median T2/FLAIR size^a^ (median, IQR)	3 (2–4)	2 (1–2)	0.23	–	0.59–2.44
Number of T2/FLAIR lesions (median, IQR)	4 (2–5)	3 (2–4)	0.45	–	0.67–3.21
Presence of SWI signal abnormality	51 (24%)	5 (4%)	**<0.001**	**7.31**	**2.83–18.90**
Location of SWI signal abnormalities					
Bilateral basal ganglia only	49 (94%)	4 (80%)			
Cerebellar nuclei only	3 (6%)	1 (20%)	0.27	4.08	0.34–48.86
Both	4 (8%)	0 (0%)			
Presence of Both T2/FLAIR and SWI abnormality	7 (3%)	2 (2%)	0.39	2.02	0.41–9.87
Presence of either T2/FLAIR and/or SWI abnormality	74 (35%)	14 (12%)	<0.001	4.08	2.18–7.63
Presence of any neuroimaging abnormality	81 (39%)	42 (35%)	0.56	1.15	0.72–1.84

IQR, interquartile range; FLAIR, fluid‐attenuated inversion recovery; SWI, susceptibility weighted imaging.

^a^ Size measured in mm. Bolded items indicate *p* <0.05.

Locations of neuroimaging abnormalities are also reported in Table 2. T2/FLAIR signal abnormalities were located diffusely, although the majority were in the frontal (40%, 12/30) and parietal lobes (37%, 11/30). SWI signal abnormalities were nearly exclusively located in the bilateral basal ganglia (96%, 49/51), although three individuals had isolated involvement of the cerebellar nuclei as well (6%, 3/51). Individuals with SWI signal abnormalities had combined basal ganglia and cerebellar involvement in four cases (8%, 4/51). Unique structures in the basal ganglia with SWI signal abnormalities included the lentiform nucleus (100%, 51/51), globus pallidus (75%, 38/51), putamen (24%, 12/51), and caudate nucleus (12%, 6/51).

Certain clinical features were associated with higher likelihood of neuroimaging abnormalities (Table 3). A history of autoimmune disease (*p =* 0.02, 95%CI: 1.11–3.51) and clinical findings of catatonia (*p* = 0.03, 95%CI: 1.05–4.74) were associated with the presence of any neuroimaging abnormalities. Prior autoimmune disease was present in 59% (30/51) of those with SWI signal abnormalities compared to 37% (11/30) of those with T2/FLAIR signal abnormalities (*p* = 0.06, OR: 2.47, 95%CI: 0.97–6.25). Catatonia was present in 96% (49/51) of those with SWI signal abnormalities compared to 70% (21/30) of those with T2/FLAIR signal abnormalities (*p* = 0.004, OR: 10.5, 95%CI: 2.09–52.8).

**Table 3:** Clinical features of individuals with DSRD in comparison to T2/FLAIR and SWI neuroimaging abnormalities.

	T2/FLAIR abnormalities (*n* = 30)	SWI abnormalities (*n* = 51)	No neuroimaging abnormalities (*n* = 123)	*p* value	95%CI
Sex					
Male (*n* = 109)	13 (43%)	30 (59%)	63 (51%)	0.79	0.61–1.89
Female (*n* = 101)	17 (57%)	21 (41%)	60 (49%)		
History of prematurity (*n* = 12)	5 (17%)	1 (2%)	4 (3%)	0.19	0.65–8.71
History of epilepsy/seizure (*n* = 5)	1 (3%)	0 (0%)	1 (1%)	0.77	0.09–24.7
History of autoimmune disease (*n* = 84)	11 (37%)	30 (59%)	42 (34%)	**0.02**	**1.11–3.51**
History of autism diagnosis^a^ (*n* = 4)	1 (3%)	1 (2%)	1 (1%)	0.36	0.28–36.6
History of thyroid disease (*n* = 58)	7 (23%)	12 (24%)	38 (31%)	0.25	0.36–1.30
Clinical features of DSRD^9^					
Altered mentals status or behavioral dysregulation	23 (77%)	38 (75%)	88 (72%)	0.55	0.64–2.29
Cognitive decline	20 (67%)	42 (82%)	93 (76%)	0.88	0.54–2.03
Developmental regression ± New autistic features	29 (97%)	51 (100%)	119 (97%)	0.38	0.29–24.5
Focal neurologic deficits and/or seizure	11 (37%)	15 (30%)	31 (25%)	0.28	0.76–2.61
Insomnia or circadian rhythm abnormalities	24 (80%)	35 (69%)	94 (76%)	0.56	0.44–1.57
Language deficits	24 (80%)	44 (86%)	92 (75%)	0.12	0.86–3.62
Movement disorder	30 (100%)	51 (100%)	123 (100%)	–	–
Catatonia	21 (70%)	49 (96%)	91 (74%)	**0.03**	**1.05–4.74**
Psychiatric symptoms	23 (77%)	36 (71%)	101 (82%)	0.12	0.29–1.14
Symptoms ≥3 years (*n* = 83)	11 (37%)	25 (49%)	47 (37%)	0.34	0.75–2.34

Clinical features of DSRD are based on International Consensus Criteria (Santoro *et al*.^10^). *p* value and 95%CI correspond to comparison between the presence of either T2 and/or SWI abnormality and no neuroimaging abnormality. Bolded items indicate *p* <0.05.

^a^ Excludes prior diagnosis of autism spectrum disorder misdiagnosed for DSRD.

Individuals with DSRD were evaluated to determine if imaging abnormalities at baseline were predictive of subsequent responses to particular therapeutic interventions (Table 4). Individuals with structural T1 abnormalities were responsive to multiple therapies, although had the highest rate of response to benzodiazepines (5/6, 83%) and the lowest rate of response to immunotherapy (1/6, 17%) when compared to those with T2/FLAIR, SWI, or no neuroimaging abnormalities. Individuals with DSRD and the presence of T2/FLAIR and/or SWI signal abnormalities were much more likely to respond to immunotherapy (*p* < 0.001, OR: 8.42. 95%CI: 3.78–18.76) and less likely to respond to benzodiazepines (*p* = 0.01, OR: 0.45, 95%CI: 0.25–0.83), antipsychotics (*p* < 0.001, OR: 0.28, 95%CI: 0.11–0.55), or electroconvulsive therapy (*p* < 0.001, OR: 0.12; 95%CI: 0.02–0.78) compared to individuals without these neuroimaging abnormalities.

**Table 4:** Therapeutic response rates by neuroimaging abnormality presence in individuals with DSRD.

	T1 abnormalities (*n* = 6)	T2/FLAIR abnormalities (*n* = 30)	SWI abnormalities (*n* = 51)	T2/FLAIR and/or SWI abnormalities (*n* = 74)	No neuroimaging abnormalities (*n* = 123)	*p* value	Odds ratio	95%CI
Benzodiazepines	5/6 (83%)	12/29 (41%)	27/50 (54%)	35/74 (47%)	69/104 (66%)	**0.01**	**0.45**	**0.25–0.83**
SSRI/SNRI	4/6 (67%)	17/30 (57%)	21/40 (53%)	38/69 (55%)	60/89 (67%)	0.11	0.59	0.31–1.13
Antipsychotics	3/6 (50%)	9/23 (39%)	12/38 (32%)	19/59 (32%)	33/50 (66%)	**<0.001**	**0.24**	**0.11–0.55**
ECT	2/3 (67%)	6/9 (67%)	8/30 (27%)	14/38 (37%)	29/35 (83%)	**<0.001**	**0.12**	**0.04–0.36**
Stimulants	0/3 (0%)	2/10 (20%)	0/10 (0%)	2/20 (10%)	8/18 (44%)	**0.03**	**0.14**	**0.02–0.78**
Immunotherapy	1/6 (17%)	20/30 (67%)	39/49 (80%)	56/70 (79%)	19/59 (32%)	**<0.001**	**8.42**	**3.78–18.76**
Steroids	–	1/4 (25%)	8/15 (53%)	9/18 (50%)	0/12 (0%)	–	–	–
IVIg	1/1 (100%)	18/25 (72%)	38/39 (97%)	54/63 (86%)	15/19 (79%)	0.48	1.60	0.43–5.92
B‐cell depletion	–	–	7/11 (64%)	7/11 (64%)	1/4 (25%)	0.21	5.25	0.40–68.95
Mycophenolate	–	1/4 (25%)	7/10 (70%)	7/13 (54%)	1/6 (17%)	0.15	5.83	0.52–64.82
Tofacitinib	–	0/1 (0%)	1/1 (100%)	1/2 (50%)	0/0 (0%)	–	–	–

*p* value and 95%CI correspond to comparison between the presence of either T2 and/or SWI abnormality and no neuroimaging abnormality. Bolded items indicate *p* <0.05.

ECT, electroconvulsive therapy; IVIg, intravenous immunoglobulin; SNRI, selective norepinephrine reuptake inhibitor; SSRI, selective serotonin reuptake inhibitor.

The location of T2/FLAIR lesions did not impact the likelihood of response to therapies (*p* = 0.67; 95%CI: 0.44–2.69). Individuals with both cerebellar and basal ganglia SWI signal abnormalities were also significantly more likely to respond to immunotherapy compared to those with DSRD who did not have these imaging abnormalities (none or T2/FLAIR only) (*p* < 0.001, OR: 6.22. 95%CI: 1.88–17.81). When directly comparing individuals with T2/FLAIR abnormalities to those with SWI abnormalities, both were equally responsive to immunotherapy overall (*p* = 0.12, OR: 0.44, 95%CI:0.16–1.22) but those with T2/FLAIR abnormalities were less responsive to intravenous immunoglobulin (IVIg), steroids, and mycophenolate than those with SWI abnormalities (*p* = 0.01, OR = 0.08, 95%CI: 0.01–0.59).

## Discussion

This study aimed to elucidate the prevalence of neuroimaging abnormalities in individuals with DSRD and evaluate their association with treatment response. While the overall rate of having any neuroimaging abnormality in individuals with and without DSRD was not statistically significant, the types of neuroimaging abnormalities in each group were markedly different. The presence of T2/FLAIR and SWI signal abnormalities were much more common in individuals with DSRD in contrast to structural abnormalities, which were more prevalent in individuals with DS alone. Importantly, the presence or absence of these imaging findings was correlated to therapeutic responses. The presence of neuroimaging abnormalities (T2/FLAIR and/or SWI) were associated with a greater than eight times higher likelihood of response to immunotherapy^7^ than in individuals with DSRD than those without these findings. Conversely, the absence of T2/FLAIR and/or SWI abnormalities was associated with higher rates of responses to benzodiazepines, antipsychotics, and ECT. This study provides evidence that neuroimaging biomarkers may be predictive of both immunotherapy and psychotropic responsiveness in individuals with DSRD.

Neuroimaging abnormalities in individuals without DSRD were much more likely to be structural in nature. Preceding diagnoses of prematurity, epilepsy, and ASD were observed frequently in this group and structural abnormalities are well established in the literature.^16^, ^17^ This higher incidence was not expected but may be partially explained by higher rates of structural abnormalities being reported in individuals with prematurity,^18^, ^19^, ^20^ epilepsy,^21^, ^22^ and ASD,^23^, ^24^ independent of the diagnosis of DS. Individuals with DSRD were not more likely to have T2/FLAIR lesions in a particular region nor more lesions than those without DSRD although there was a nonsignificant trend toward a greater lesion size in the DSRD cohort. The lack of differentiation between groups with this particular neuroimaging finding is unclear as those with DSRD and T2/FLAIR signal abnormalities respond to immunotherapy. T2/FLAIR signal abnormalities are appreciated in individuals with DS over the life span in persons and may have many causes.^25^ It is possible that T2/FLAIR abnormalities may reflect both inflammatory and noninflammatory etiologies or be a process driven by the SWI signal which overlapped with T2/FLAIR abnormalities in many cases.

SWI abnormalities were strikingly different between groups and correlated closely with comorbid autoimmune disease and the presence of catatonia, both being common findings in individuals with DSRD.^5^, ^7^ The exact pathologic mechanism by which these abnormalities emerge is unknown, although higher rates of comorbid autoimmune disease in those with these findings could indicate a potential role of chronic immune activation. From a clinical perspective, the association between SWI imaging abnormalities in the basal ganglia and the presence of catatonia are logical from a localization standpoint as the basal ganglia is the movement generation center of the brain. This is a particularly important finding given high rates of catatonia in DSRD and the fact that it may be under‐recognized as an initial symptom at the time of presentation,^26^ which could alert clinicians to underlying autoimmune neurologic phenomenon.^27^, ^28^

Biomarkers remain an area of great interest in the diagnosis and treatment of individuals with DSRD. While neuroimaging abnormalities are not necessarily unique or pathognomonic for this condition, this study revealed a noticeably increased likelihood of response to immunotherapy when these findings are present and an increased likelihood of responding to benzodiazepines, antipsychotics, and ECT when absent. While the exact etiology of this association is unclear, one hypothesis is that T2/FLAIR and/or SWI signal abnormalities could be caused by an underlying inflammatory pathologic mechanism that is augmented with immunotherapy. This is supported by the higher rates of neuroimaging abnormalities observed in those with both DSRD and comorbid autoimmune diseases in our cohort. Multiple smaller studies have revealed that the incidence of basal ganglia mineralization (which would manifest on MRI as SWI signal abnormalities) in persons with DS increases with age.^29^, ^30^ Interestingly, these findings have also been reported in the periphery indicating a more systemic etiology,^31^, ^32^, ^33^, ^34^ and are potentially related to dysregulated interferon response which has been extensively reported in individuals with DS.^35^ In many ways, it is thus unsurprising that in a condition associated with elevated interferon signaling as extended interferon exposure over time could lead to these findings, with higher rates being observed over time. This phenomenon is also reported in a naturally aging population and thus must be viewed in this context as well.^36^

Immunotherapy responsiveness in individuals with DSRD in the context of neuroimaging abnormalities will be increasingly important to consider as therapeutic protocols for this condition evolve. While the diagnosis of DSRD remains one of exclusion and is clinical in nature, the presence of neuroimaging biomarkers during diagnostic workup may provide guidance for initial interventions. This is particularly important given that the absence of neuroimaging abnormalities and the presence of structural T1 abnormalities predicted a higher response rate to benzodiazepines, antipsychotics, stimulants, and ECT. The multiple therapeutics that treat DSRD both with and without association to neuroimaging biomarkers, highlight the heterogenous nature of the condition and potential for multifactorial pathophysiologic mechanism. The authors hope this data will lead to the prospective development of treatment profiles for individuals with DSRD and guide more personalized approaches to therapeutic interventions. Further, prospective studies evaluating longitudinal changes in T2/FLAIR and SWI abnormalities will also be of great utility as would metabolic imaging such as FDG‐PET which could provide another objective method by which to differentiate DSRD from other conditions that could overlap in symptoms such as AD.

There are multiple limitations to this study which should be considered when interpreting the results. This study is retrospective with data sequence capture being heterogenous and at different time points in the disease course. While the study utilized a strict quality control measure and relied on blinded independent neuroradiologists for study interpretation, many individuals were excluded from review. The inter‐relator reliability was high in this study which could have been secondary to neuroradiologists being asked to comment on the presence or absence of neuroimaging abnormalities (as opposed to detailing volumetric abnormalities). Most individuals in this study were evaluated at academic medical centers which introduces both severity bias and selection bias in both the experimental and control groups. As such, it is possible that higher rates of neuroimaging abnormalities could be present in both groups compared to real‐world populations of persons with DS. An additional effect of this may have been realized in the high rates of structural abnormalities observed in the control cohort as neuroimaging is not considered standard of care of many individuals with DS who have neurologic symptoms. In addition, the time between symptom onset and neuroimaging was variable in this cohort, potentially introducing a higher likelihood of imaging abnormality capture when symptoms were present for longer periods of time. A mixed effect model was used to reduce the impact of this heterogeneity when interpreting data. As interferon‐driven mineralization is more likely over extended periods of time, this could have influenced the likelihood of capture of SWI imaging abnormalities. Strict inclusion/exclusion criteria were applied to this study from a neuroimaging standard perspective, although sequences were of nonuniform quantity and quality, making advanced testing such as volumetric analysis very challenging. Neuroimaging abnormalities can be influenced by multiple factors and a major limitation of this study is that nearly all patients never had prior neuroimaging performed, so it is difficult to assess if DSRD was truly the cause of these findings. As such, this study highlights the importance of prospective standardized quantitative neuroimaging (particularly SWI‐based) studies in individuals with DSRD which can provide more objective data than reported in this study. The authors compared this data to clinical outcomes as a means of assessing if these findings were meaningful, but this is not a confirmatory method of determining pathogenicity. Finally, clinical response was evaluated using validated metrics such as the NPI‐Q, BFCRS, 25FW, and CGI with an a priori cutoff of greater than 50% improvement to determine clinical response. This was an arbitrary cutoff point set for the purposes of this study, although future, prospective studies will likely elucidate the meaningfulness of these outcome measures. In addition, not all therapeutic class responses could be tabulated for each patient as therapeutic trials before the obtainment of the NPI‐Q, BFCRS, 25FW, and CGI were excluded due to the lack of ability to objectively assess response.

In summary, individuals with DSRD have a higher than previously reported prevalence of neuroimaging abnormalities when compared to age‐ and sex‐matched individuals with DS who had undergone neuroimaging. While most individuals with DSRD will not have imaging abnormalities, those who do have a greater than eight times higher likelihood of response to immunotherapy. This is the first study to identify a correlation between clinical and radiographic features in individuals with DSRD, identifying a potential first step in the use of neurodiagnostic biomarkers for DSRD. Future prospective controlled studies into the mechanisms underlying these imaging findings and associated immunotherapy responsiveness will be crucially important.

## Author Contributions

JDS and MSR conceptualized and designed the study, analyzed data, drafted significant portions of the article, and reviewed the article for intellectual content. MMK, SJ, LN, NKB, BNV, RK, LP, MAM, ALR, RAF, NTB, SLS, and CF assisted with data collection, data analysis, drafted portions of the article, and reviewed for intellectual content. BT and KWY assisted with data analysis and interpretation and reviewed the article for intellectual content. GW and JME helped conceptualize the study and interpret data and reviewed the article for intellectual content.

## Conflict of Interest

Dr. Jonathan D. Santoro has received consulting funds from Cycle Pharma and UCB on topics relating to multiple sclerosis and myelin oligodendrocyte glycoprotein‐related disorder. Dr. Espinosa is the executive director of the Linda Crnic Institute for Down Syndrome at the University of Colorado which receives funds from the Global Down Syndrome Foundation.

## Data Availability

Anonymized data are available to qualified researchers upon request to the corresponding author and IRB approval.

## References

[1] de Graaf G , Buckley F , Skotko BG . Estimates of the live births, natural losses, and elective terminations with Down syndrome in the United States. Am J Med Genet A. 2015;167:756‐767. 10.1002/ajmg.a.37001 25822844

[2] Antonarakis SE , Skotko BG , Rafii MS , et al. Down syndrome. Nat Rev Dis Primers. 2020;6:9. 32029743 10.1038/s41572-019-0143-7 PMC8428796

[3] Worley G , Crissman BG , Cadogan E , Milleson C , Adkins DW , Kishnani PS . Down syndrome disintegrative disorder: new‐onset autistic regression, dementia, and insomnia in older children and adolescents with Down syndrome. J Child Neurol. 2015;30:1147‐1152. 25367918 10.1177/0883073814554654

[4] Mircher C , Cieuta‐Walti C , Marey I , et al. Acute regression in young people with Down syndrome. Brain Sci. 2017;7:7. 10.3390/brainsci7060057 PMC5483630 28555009

[5] Santoro SL , Baumer NT , Cornacchia M , et al. Unexplained regression in Down syndrome: management of 51 patients in an international patient database. Am J Med Genet A. 2022;188:3049‐3062. 35924793 10.1002/ajmg.a.62922

[6] Santoro SL , Cannon S , Capone G , et al. Unexplained regression in Down syndrome: 35 cases from an international Down syndrome database. Genet Med. 2020;22:767‐776. 31767984 10.1038/s41436-019-0706-8

[7] Santoro JD , Partridge R , Tanna R , et al. Evidence of neuroinflammation and immunotherapy responsiveness in individuals with down syndrome regression disorder. J Neurodev Disord. 2022;14:35. 35659536 10.1186/s11689-022-09446-w PMC9164321

[8] Ghaziuddin N , Nassiri A , Miles JH . Catatonia in Down syndrome; a treatable cause of regression. Neuropsychiatr Dis Treat. 2015;11:941‐949. 25897230 10.2147/NDT.S77307 PMC4396650

[9] Gregory A , Wilson JL , Hogarth P , Hayflick SJ . Abnormal brain iron accumulation is a rare finding in Down syndrome regression disorder. Pediatr Neurol. 2023;138:1‐4. 36270151 10.1016/j.pediatrneurol.2022.09.002

[10] Santoro JD , Patel L , Kammeyer R , et al. Assessment and diagnosis of Down syndrome regression disorder: international expert consensus. Front Neurol. 2022;13:940175. 35911905 10.3389/fneur.2022.940175 PMC9335003

[11] Kaufer DI , Cummings JL , Ketchel P , et al. Validation of the NPI‐Q, a brief clinical form of the neuropsychiatric inventory. J Neuropsychiatry Clin Neurosci. 2000;12:233‐239. 11001602 10.1176/jnp.12.2.233

[12] Miles JH , Takahashi N , Muckerman J , Nowell KP , Ithman M . Catatonia in Down syndrome: systematic approach to diagnosis, treatment and outcome assessment based on a case series of seven patients. Neuropsychiatr Dis Treat. 2019;15:2723‐2741. 31571888 10.2147/NDT.S210613 PMC6759875

[13] Sienaert P , Rooseleer J , De Fruyt J . Measuring catatonia: a systematic review of rating scales. J Affect Disord. 2011;135:1‐9. 21420736 10.1016/j.jad.2011.02.012

[14] Goldman MD , Motl RW , Scagnelli J , Pula JH , Sosnoff JJ , Cadavid D . Clinically meaningful performance benchmarks in MS: timed 25‐foot walk and the real world. Neurology. 2013;81:1856‐1863. 24174581 10.1212/01.wnl.0000436065.97642.d2 PMC3821712

[15] Toolan C , Holbrook A , Schlink A , Shire S , Brady N , Kasari C . Using the clinical global impression scale to assess social communication change in minimally verbal children with autism spectrum disorder. Autism Res. 2022;15:284‐295. 34800004 10.1002/aur.2638 PMC8821201

[16] Shiohama T , Levman J , Baumer N , Takahashi E . Structural magnetic resonance imaging‐based brain morphology study in infants and toddlers with Down syndrome: the effect of comorbidities. Pediatr Neurol. 2019;100:67‐73. 31036426 10.1016/j.pediatrneurol.2019.03.015 PMC6755072

[17] Trowbridge SK , Yuskaitis CJ , Baumer N , Libenson M , Prabhu SP , Harini C . Brain MRI abnormalities in patients with infantile spasms and Down syndrome. Epilepsy Behav. 2019;92:57‐60. 30616066 10.1016/j.yebeh.2018.12.013

[18] Ji W , Li G , Jiang F , et al. Preterm birth associated alterations in brain structure, cognitive functioning and behavior in children from the ABCD dataset. Psychol Med. 2024;54:409‐418. 37365781 10.1017/S0033291723001757

[19] Schmitz‐Koep B , Menegaux A , Gaser C , et al. Altered gray matter cortical and subcortical T1‐weighted/T2‐weighted ratio in premature‐born adults. Biol Psychiatry Cogn Neurosci Neuroimaging. 2023;8:495‐504. 35276405 10.1016/j.bpsc.2022.02.013

[20] Nosarti C , Nam KW , Walshe M , et al. Preterm birth and structural brain alterations in early adulthood. Neuroimage Clin. 2014;6:180‐191. 25379430 10.1016/j.nicl.2014.08.005 PMC4215396

[21] Grünewald RA , Farrow T , Vaughan P , Rittey CD , Mundy J . A magnetic resonance study of complicated early childhood convulsion. J Neurol Neurosurg Psychiatry. 2001;71:638‐642. 11606676 10.1136/jnnp.71.5.638 PMC1737589

[22] Stern T , Kornreich L , Goldberg H . Yield of brain magnetic resonance imaging in epilepsy diagnosis from 1998 to 2020: a large retrospective cohort study. Neuropediatrics. 2022;53:15‐19. 34327696 10.1055/s-0041-1732325

[23] Xu MX , Ju XD . Abnormal brain structure is associated with social and communication deficits in children with autism Spectrum disorder: a voxel‐based morphometry analysis. Brain Sci. 2023;13:13. 10.3390/brainsci13050779 PMC10216141 37239251

[24] Courchesne E , Karns CM , Davis HR , et al. Unusual brain growth patterns in early life in patients with autistic disorder: an MRI study. Neurology. 2001;57:245‐254. 11468308 10.1212/wnl.57.2.245

[25] Roth GM , Sun B , Greensite FS , Lott IT , Dietrich RB . Premature aging in persons with Down syndrome: MR findings. AJNR Am J Neuroradiol. 1996;17:1283‐1289. 8871713 PMC8338525

[26] Jakubowicz B , Baptista A , Ravel A , et al. Catatonia in Down's syndrome: an under‐recognized syndrome during regression. Schizophr Res. 2023;261:107‐109. 37716203 10.1016/j.schres.2023.09.011

[27] Minamisawa Y , Sato M , Saito Y , et al. Case report: evolution of catatonic mutism and psychotic symptoms in an adolescent with Down syndrome: transition from Down syndrome disintegrative disorder to anti‐N‐methyl‐D‐aspartate receptor encephalitis. Front Neurol. 2023;14:1200541. 37360353 10.3389/fneur.2023.1200541 PMC10288866

[28] Helene V , Patrick V , Boel de P , Gini B , Bruno B , Rudy VC . Hashimoto encephalopathy and antibodies against dimethylargininase‐1: a rare cause of cognitive decline in a pediatric Down's syndrome patient. Clin Neurol Neurosurg. 2011;113:678‐679. 21570763 10.1016/j.clineuro.2011.04.004

[29] Arai Y , Yoshihara S , Iinuma K . Brain CT studies in 26 cases of aged patients with Down syndrome. No To Hattatsu. 1995;27:17‐22. 7873245

[30] Wegiel J , Kuchna I , Wisniewski T , et al. Vascular fibrosis and calcification in the hippocampus in aging, Alzheimer disease, and Down syndrome. Acta Neuropathol. 2002;103:333‐343. 11904752 10.1007/s00401-001-0471-y

[31] Delaporte E , Gosselin P , Catteau B , Nuyts JP , Piette F , Bergoend H . Perforating milia‐like idiopathic calcinosis of the extremities in Down syndrome. Ann Dermatol Venereol. 1997;124:159‐161. 9740827

[32] Schepis C , Siragusa M , Palazzo R , Batolo D , Romano C . Milia‐like idiopathic calcinosis cutis: an unusual dermatosis associated with Down syndrome. Br J Dermatol. 1996;134:143‐146. 8745902

[33] Kanzaki T , Nakajima M . Milialike idiopathic calcinosis cutis and syringoma in Down's syndrome. J Dermatol. 1991;18:616‐618. 1838753 10.1111/j.1346-8138.1991.tb03143.x

[34] Schepis C , Siragusa M , Palazzo R , Batolo D , Romano C . Perforating milia‐like idiopathic calcinosis cutis and periorbital syringomas in a girl with Down syndrome. Pediatr Dermatol. 1994;11:258‐260. 7971561 10.1111/j.1525-1470.1994.tb00598.x

[35] Waugh KA , Araya P , Pandey A , et al. Mass cytometry reveals global immune remodeling with multi‐lineage hypersensitivity to type I interferon in Down syndrome. Cell Rep. 2019;29:1893‐1908.e1894. 31722205 10.1016/j.celrep.2019.10.038 PMC6871766

[36] Harder SL , Hopp KM , Ward H , Neglio H , Gitlin J , Kido D . Mineralization of the deep gray matter with age: a retrospective review with susceptibility‐weighted MR imaging. AJNR Am J Neuroradiol. 2008;29:176‐183. 17989376 10.3174/ajnr.A0770 PMC8119097

